# Functional C‐TERMINALLY ENCODED PEPTIDE (CEP) plant hormone domains evolved *de novo* in the plant parasite *Rotylenchulus reniformis*

**DOI:** 10.1111/mpp.12402

**Published:** 2016-06-06

**Authors:** Sebastian Eves‐Van Den Akker, Catherine J. Lilley, Hazijah B. Yusup, John T. Jones, Peter E. Urwin

**Affiliations:** ^1^ Division of Plant Sciences College of Life Sciences, University of Dundee Dundee DD1 5EH UK; ^2^ Centre for Plant Sciences University of Leeds Leeds LS2 9JT UK; ^3^ Cell and Molecular Sciences Group, Dundee Effector Consortium The James Hutton Institute Invergowrie Dundee DD2 5DA UK; ^4^ School of Biology University of St Andrews North Haugh, St Andrews KY16 9TZ UK; ^5^ Present address: Universiti Teknologi MARA Sarawak 94300 Kota Samarahan Sarawak Malaysia

**Keywords:** effector, evolution, plant‐parasitic nematode, plant peptide hormone

## Abstract

Sedentary plant‐parasitic nematodes (PPNs) induce and maintain an intimate relationship with their host, stimulating cells adjacent to root vascular tissue to re‐differentiate into unique and metabolically active ‘feeding sites’. The interaction between PPNs and their host is mediated by nematode effectors. We describe the discovery of a large and diverse family of effector genes, encoding C‐TERMINALLY ENCODED PEPTIDE (CEP) plant hormone mimics (RrCEPs), in the syncytia‐forming plant parasite *Rotylenchulus reniformis*. The particular attributes of RrCEPs distinguish them from all other CEPs, regardless of origin. Together with the distant phylogenetic relationship of *R. reniformis* to the only other CEP‐encoding nematode genus identified to date (*Meloidogyne*), this suggests that CEPs probably evolved *de novo* in *R. reniformis*. We have characterized the first member of this large gene family (*RrCEP1*), demonstrating its significant up‐regulation during the plant–nematode interaction and expression in the effector‐producing pharyngeal gland cell. All internal CEP domains of multi‐domain RrCEPs are followed by di‐basic residues, suggesting a mechanism for cleavage. A synthetic peptide corresponding to RrCEP1 domain 1 is biologically active and capable of up‐regulating plant nitrate transporter (*AtNRT2.1*) expression, whilst simultaneously reducing primary root elongation. When a non‐CEP‐containing, syncytia‐forming PPN species (*Heterodera schachtii*) infects *Arabidopsis* in a CEP‐rich environment, a smaller feeding site is produced. We hypothesize that CEPs of *R. reniformis* represent a two‐fold adaptation to sustained biotrophy in this species: (i) increasing host nitrate uptake, whilst (ii) limiting the size of the syncytial feeding site produced.

## Introduction

Sedentary plant‐parasitic nematodes (PPNs) induce and maintain an intimate relationship with their host. These obligate biotrophs stimulate root cells to re‐differentiate into unique and metabolically active ‘feeding sites’, from which they feed exclusively for a number of weeks until their life cycle is completed. On selection of the initial feeding cell, the motile nematodes become sedentary and are thus committed to that site. They must maintain the biotrophic interaction for several weeks; if the plant dies or the feeding site ceases to function and to provide nutrients, the nematode will also die. This obligate dependence on host survival, which, in some cases, can extend over many nematode generations, is a strong selection pressure against killing the host.

There are several genera of sedentary PPNs that impose varying degrees of host damage, induce feeding sites of different ontogeny and in which parasitism has evolved independently (van Megen *et al*., 2009). The multinucleate feeding sites of the most widely studied sedentary PPNs can be divided into two general classes: (i) the cluster of individual giant cells of the root‐knot nematodes, each formed from a single cell by multiple rounds of mitosis in the absence of cytokinesis (de Almeida Engler *et al*., 1999); (ii) the syncytial feeding sites of the cyst, reniform and false‐root‐knot nematodes, formed by partial cell wall dissolution and subsequent protoplast fusion of multiple adjacent cells (Grymaszewska and Golinowski, 1998; Holtmann *et al*., 2000; Jones and Payne, 1977; Rahman Razak and Evans, 1976; Robinson *et al*., 1998; Sobczak and Golinowski, 2011). Syncytia of cyst nematodes are typically initiated from a single pericycle, procambial or inner cortical cell. Increased cytoplasmic density and proliferation of organelles are accompanied by widening of existing plasmodesmata, followed by local dissolution of the cell walls between the syncytium and neighbouring cells and fusion of protoplasts. In the early stages of feeding site development, the syncytium grows by continually incorporating neighbouring cells which have been stimulated to divide (Sobczak and Golinowski, 2011). The syncytia of reniform and false‐root‐knot nematodes form by similar cell wall dissolution and share many of the same characteristics. For *Rotylenchulus reniformis*, the initial syncytial cell is typically an endodermal cell (Robinson *et al*., 1998) and the feeding site subsequently extends around the root as a single, curved cell layer (Jones and Dropkin, 1975).

Much of the work to understand the molecular basis of the relationship between parasites and their hosts focuses on parasite proteins secreted during the interaction, termed ‘effectors’. Although effectors of PPNs can originate from a range of tissues, the vast majority are produced in pharyngeal gland cells and are delivered into the host via a hollow, protractible stylet. A number of classes of effectors have been described to date (reviewed in Hewezi, 2015; Mitchum *et al*., 2013). The most relevant to this work are those which ‘mimic’ post‐translationally modified plant peptide hormones in both sequence and function (Bobay *et al*., 2013; Mitchum *et al*., 2012; Wang *et al*., 2005). Two such classes have been identified to date. The first (Wang *et al*., 2005) is the CLAVATA3/ESR (CLE)‐like peptides which are present in all sedentary PPNs (Mitchum *et al*., 2012), regardless of their origin of parasitism. More recently, genes encoding C‐TERMINALLY ENCODED PEPTIDES (CEPs) have been identified in a single lineage, the giant cell‐forming root‐knot nematodes (Bird *et al*., 2015; Bobay *et al*., 2013), although neither the gland cell expression nor biological activity of the peptides has been reported.

CEPs were discovered in plants relatively recently (Ohyama *et al*., 2008), and are an ancient peptide hormone common to all vascular land plants. More than 900 CEP genes have been identified to date (Ogilvie *et al*., 2014; Roberts *et al*., 2013), although only those from *Arabidopsis* (Delay *et al*., 2013; Ohyama *et al*., 2008; Roberts *et al*., 2013; Tabata *et al*., 2014) and *Medicago truncatula* (Bobay *et al*., 2013; Imin *et al*., 2013; Mohd‐Radzman *et al*., 2015) have been characterized in detail. CEP precursor proteins contain a signal peptide for secretion, followed by at least one CEP domain, and are processed in the apoplast into biologically active 15‐amino‐acid peptides. These short peptides are most typically modified with hydroxyprolines in positions 4 and 11 (Ohyama *et al*., 2008), although variants with hydroxylation of the proline (Pro) at position 7 and/or arabinosylation of Pro‐11 have been identified (Mohd‐Radzman *et al*., 2015). The use of synthetic functional CEP peptides, corresponding to domains within genes of interest, has allowed rapid advances in the field (Bobay *et al*., 2013; Delay *et al*., 2013; Imin *et al*., 2013; Ohyama *et al*., 2008; Tabata *et al*., 2014). The current paradigm suggests that *CEP* genes are up‐regulated by nitrogen starvation (Imin *et al*., 2013) and that the active peptides move in the xylem to the leaves, where they initiate an as yet unidentified descending signal which results in increased and compensatory up‐regulation of nitrate transporters on a whole‐root scale. Two *Arabidopsis* leucine‐rich repeat receptor kinases (LRR‐RKs; CEPR1 and CEPR2) have been identified as the shoot‐expressed receptors for several CEPs (Tabata *et al*., 2014). CEPs are clearly multifunctional, as one of their first described roles was the modulation of plant architecture (Ohyama *et al*., 2008). Over‐expression of *CEP* genes or exogenous application of CEP domain peptides to *Arabidopsis* results in suppression of the rate of root cell division, as evidenced by reduced primary root elongation (Delay *et al*., 2013). This can be rationalized by systemic up‐regulation of nitrate transporters, presumably as an adaptive local response to reduce plant growth where nitrate is limiting, and thus where *CEP*s are up‐regulated. The function of CEPs in PPNs remains unclear. Over‐expression of MtCEP1 or exogenous application of the CEP domain peptide to *Md. truncatula* produces periodic, circumferential root swellings which are phenotypically similar to the galls induced in this plant by the root‐knot nematode *Meloidogyne hapla* (Imin *et al*., 2013); however, similar activity has not been described for the *M. hapla* peptides.

We have identified a large family of *CEP* genes in the reniform nematode *R. reniformis*. This sedentary semi‐endoparasite, which has a host range encompassing more than 300 plant species, induces a syncytial feeding site and does not cause galling of infected roots. Second‐stage juveniles (J2s) hatch from eggs in the soil and then develop to vermiform adult females and males via non‐motile, non‐feeding J3 and J4 stages. Only the female nematode invades the host root and becomes sedentary, with the posterior body remaining outside and subsequently swelling to a kidney shape (Robinson *et al*., 1998). We present evidence to suggest that CEPs in *R. reniformis* originated independently from those in both plants and the root‐knot nematodes. *Rotylenchulus reniformis* CEPs (RrCEPs) are characterized by unique features; unlike CEPs from all other organisms, those cloned from *R. reniformis* contain one intron per domain sequence, regardless of the number of tandem domains that are present. We characterize one member of the *RrCEP* family in detail and demonstrate that it is highly up‐regulated during the biotrophic infection phase of the life cycle and expressed in the effector‐producing pharyngeal gland cell. We show that this gene, *RrCEP1*, encodes a functional CEP domain which significantly up‐regulates a host nitrate transporter. This domain also inhibits root elongation and limits feeding site expansion for a non‐CEP‐containing, syncytial‐forming cyst nematode. CEP effectors of *R. reniformis* may therefore represent a two‐fold adaptation to sustained biotrophy by: (i) increasing host nitrate uptake, whilst (ii) limiting the size of the syncytial feeding site produced.

## Results and Discussion

### *Rotylenchulus reniformis* contains a large and diverse family of CEP genes

**Figure 1:**
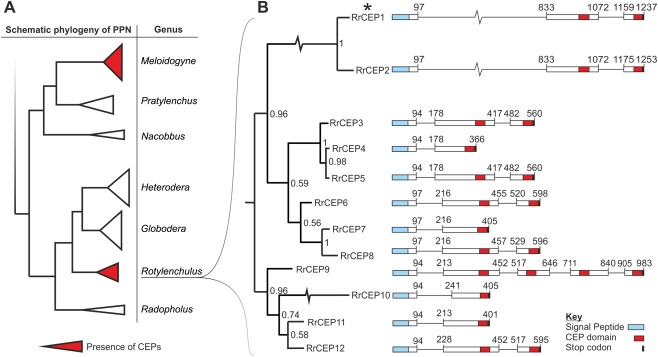
Phylogenetic analysis and genetic structure of the C‐terminally‐encoded peptide (CEP) gene family in *Rotylenchulus reniformis*. (A) Schematic diagram of the local phylogenetic neighbourhood of sedentary plant‐parasitic nematodes, including the only two CEP‐containing genera. *Rotylenchulus reniformis* has a distant phylogenetic relationship and an independent origin of biotrophic parasitism to the only other CEP‐containing nematodes, with many intermediate species that lack CEPs (van Megen *et al*., 2009). (B) Using a mid‐point re‐routed Bayesian phylogeny, all CEP sequences cloned to date from *R. reniformis* genomic DNA can be grouped into several distinct clades based on an alignment of complete genomic sequences. A representative genetic structure of the sequences in each clade identifies a large variation in CEP domain number and arrangement. Despite the diversity, CEP domain motifs are highly similar between clades. Unlike CEPs from all other organisms (Bobay *et al*., 1994; Delay *et al*., 2013), those of *R. reniformis* all contain at least one intron. Numbers correspond to base position; signal peptides (blue), CEP domains (red) and stop codons (black) are indicated. *Indicates RrCEP1 for further study.

Transcripts containing CEP‐like domains, identified in unpublished *R. reniformis* next‐generation sequencing (NGS) data (ERA PRJEB8325 and SRR949271), were used to design primers to amplify sequences of interest. Using a primer pair targeting a single CEP‐like gene, multiple polymerase chain reaction (PCR) products were generated from genomic DNA; these were cloned and sequenced. Cloned genomic sequences which encoded complete open reading frames, were unique at the protein level (or, where identical, contained considerably different introns) and were different in total gene length were deemed to arise from unique loci and used to construct a preliminary phylogeny of the gene family (Fig. 1 and Dryad accession doi:10.5061/dryad.q8h75). The level of sequence diversity within Fig. 1 is higher than that likely to arise as a result of allelic variation, and thus the 12 cloned genomic sequences included were named sequentially.

**Figure 2:**
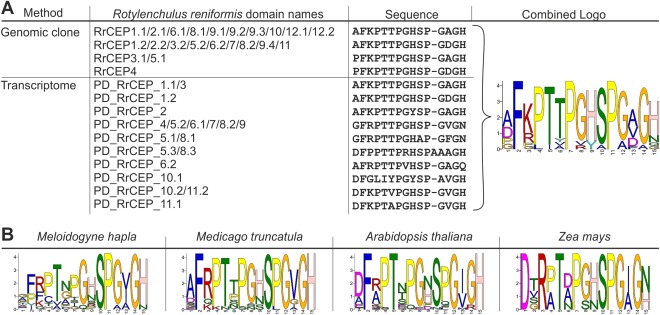
C‐terminally‐encoded peptide (CEP) domain alignments between kingdoms. (A) A combination of all unique *Rotylenchulus reniformis* CEP domains from the 12 cloned genes displayed in Fig. 1 and all unique putative domains (PDs) present in a *de novo* transcriptome assembly. According to the grouping system proposed by both Delay *et al*. (2013) and Roberts *et al*. (2013), all *R. reniformis* CEPs conform to group I. (B) For comparison, logo plots are shown of all unique CEP domains from the root‐knot nematode *Meloidogyne hapla*, and all group I CEP domains from the dicotyledons *Medicago truncatula* and *Arabidopsis thaliana* and the monocotyledon *Zea mays*.

Analysis of the *R. reniformis* transcriptomic data showed that the full complement of the cloned CEP sequences is not represented in the assembled transcriptome and, similarly, that not all sequences present in the transcriptome were cloned. Given this disparity, it is therefore likely that we have identified only a subset of what is a large and diverse family of CEP‐encoding genes in *R. reniformis*. Adopting a conservative approach, for their first identification in *R. reniformis*, we focus on only the cloned genes and their deduced amino acid sequences with the aim of characterizing their function and providing a basis for further study. As for plant and other nematode CEPs, all *R. reniformis* CEP genes (*RrCEP*s) encode a signal peptide, followed by at least one CEP domain. According to the grouping system proposed by both Delay *et al*. (2013) and Roberts *et al*. (2013), all RrCEPs conform to group I. By comparing group I CEP domain sequences between kingdoms (Fig. 2), those of *R. reniformis* conform well to the expected characteristics indicative of bona fide CEPs.

### RrCEPs are additionally characterized by unique attributes and probably evolved *de novo* in *R. reniformis*

CEP precursor proteins in *R. reniformis* can contain several CEP domains in tandem (*n* ^max^ = 4), where the final domain is followed directly by a stop codon (Fig. 1). Although multiple domains are common in plant CEPs, and indeed occur in other nematode peptide hormone mimics, e.g. CLEs of the potato cyst nematode *Globodera rostochiensis* (Lu *et al*., 2009), they are unique among nematode CEPs. All CEP genes cloned from *R. reniformis* contain one intron per domain sequence, regardless of the number of tandem domains. Introns range in size from <100 to >700 base pairs (Fig. 1). This is highly unusual as all of the other several hundred CEPs identified to date, from plant or animal origin, are encoded on a single exon (Bobay *et al*., 2013; Delay *et al*., 2013; Ogilvie *et al*., 2014). The biological significance of introns in *RrCEPs* (particularly in multi‐domain CEP effectors), or of single exon genes in every other genus, is unclear. There is no evidence that the intron structure of *R. reniformis* CEPs introduces additional variation through alternative splicing.

An insight into the genomic organization of *RrCEP*s was gained during the cloning and sequencing process. A genomic DNA fragment was cloned that encoded two RrCEPs in tandem. The two CEP gene models, in the same orientation, overlap, yet exist in different reading frames (Fig. S1, see Supporting Information). Precedent exists for this type of genomic organization in the bacterial EcoKI DNA methyltransferase (Roberts *et al*., 2012). This trimeric protein comprises two modification subunits (M) and one sequence specificity subunit (S). The 3′ end of the gene encoding the M subunit overlaps the 5′ end of the S subunit by one nucleotide where translation from the two different open reading frames is translationally coupled (Roberts *et al*., 2012). Further investigation will be required to determine whether these RrCEPs are similarly translationally coupled, and whether or not both are produced as full‐length functional proteins. These genes are identical in deduced coding sequence, with the exception that the first does not contain a full‐length CEP domain. The second is already represented in the phylogeny at 100% amino acid identity; thus, neither gene was additionally included in our analysis.

With the exception of the 15‐amino‐acid CEP domain itself, *R. reniformis* CEPs share no sequence similarity with any other CEPs from plants or animals. Despite the inherent difficulty associated with the limited phylogenetic signal and functional sequence constraints of CEPs, *R. reniformis* CEPs probably arose independently of other plant and animal CEPs. This is supported by the lack of sequence similarity outside of the CEP domain, the unusual genetic structure seen within *R. reniformis* and the fact that the only other known CEP‐encoding nematode genus (*Meloidogyne*) is distantly related to *R. reniformis* with an independent origin of biotrophy (van Megen *et al*., 2009). These two genera are separated by many CEP‐absent intermediate species. Contrary to CLEs, there is no evidence of CEP‐like sequences in available cyst nematode genomes (to which *R. reniformis* is basal), suggesting that these sequences arose after the split from the last common ancestor between *R. reniformis* and cyst nematodes.

### *Rotylenchulus reniformis CEP1* is highly expressed in a large secretory pharyngeal gland cell during plant–nematode biotrophic interactions

**Figure 3:**
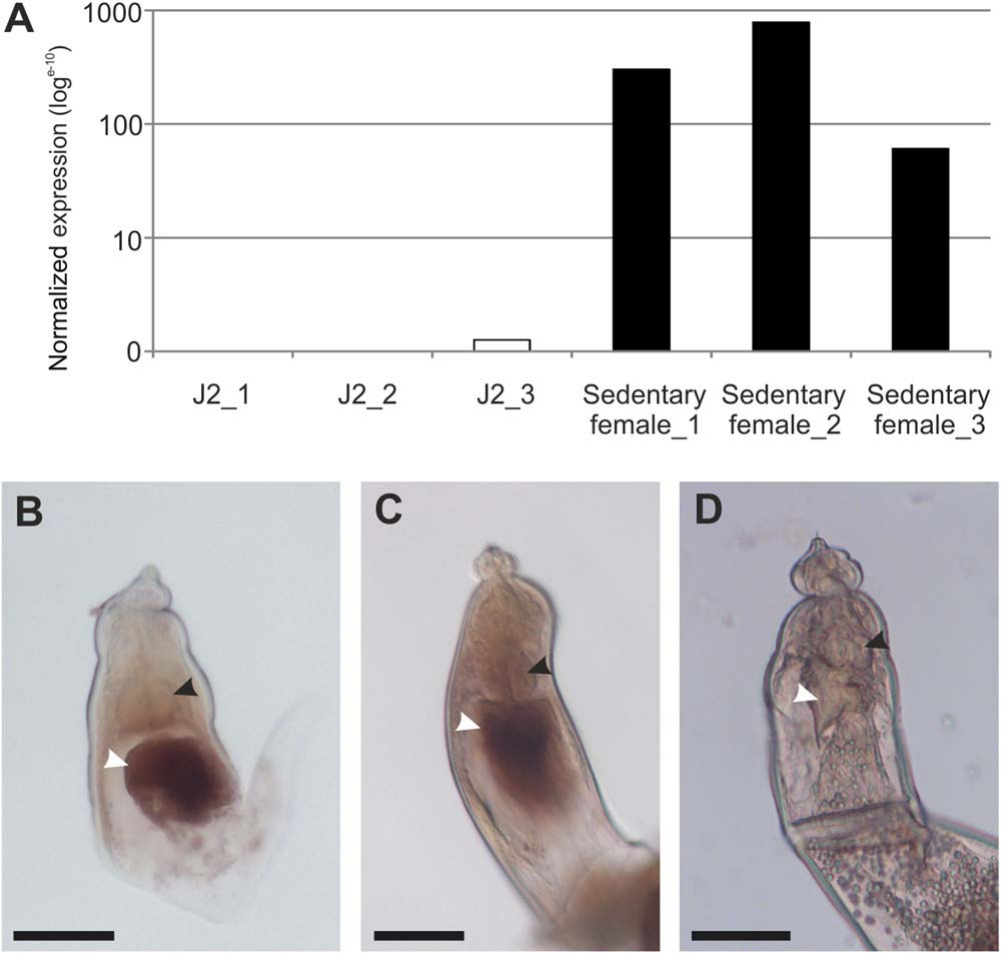
*Rotylenchulus reniformis* C‐terminally‐encoded peptide (CEP) temporal and spatial expression. (A) *RrCEP1* is significantly up‐regulated during the sedentary parasitic phase of the life cycle compared with the second‐stage juvenile (*n* = 3, *P* < 0.001). Normalized expression data from each of three independent RNAseq libraries is shown for juvenile and sedentary female stages. (B, C) *In situ* hybridization identifies *R. reniformis CEP1* expression in a large secretory gland cell (white arrow) posterior to the metacorpal bulb (black arrow) of mature females. (D) No such staining is observed with the negative control *in situ* hybridization probe. Scale bars indicate 40 μm.

As transcripts corresponding to the deduced coding sequence of *RrCEP1* were present in the transcriptome assembly, this gene was studied further. *RrCEP1* is highly up‐regulated during the biotrophic phase of the plant–nematode interaction (Fig. 3A, *P* < 0.001). Using a complementary digoxigenin‐labelled DNA probe for *in situ* hybridization (de Boer *et al*., 1998), the *RrCEP1* transcript was localized in the single large secretory pharyngeal gland cell (Fig. 3B,C). No such staining pattern was seen with the non‐complementary negative control DNA probe (Fig. 3D). Contrary to other PPNs, adult female *R. reniformis* apparently contain only a single large pharyngeal gland cell (Robinson *et al*., 1998), consistent with the images presented here. This indicates that the protein encoded by *RrCEP1* is likely to be secreted *in planta* during the biotrophic interaction.

Plant peptide hormones function in the apoplast, where they bind to transmembrane LRR‐RKs (Matsubayashi, 2014). The exact site of delivery *in planta* for *R. reniformis* CEPs is at present unclear; the gland cells of PPNs deliver proteins both inside the feeding site cytoplasm (Replogle *et al*., 2011) and directly into the apoplast (Vieira *et al*., 2011). CLE peptide precursors of the plant‐parasitic cyst nematode *Heterodera glycines* are apparently delivered into the feeding site cytoplasm, where they can be detected by immunolocalization (Wang *et al*., 2010). The canonical signal peptide is cleaved on translocation into the gland cell endoplasmic reticulum within the nematode, but the CLE pro‐peptide is then secreted from the plant cell into the apoplast by a post‐translational trafficking mechanism (Wang *et al*., 2010). It is not clear how this functions, but a putative second ‘cryptic’ signal peptide, present in the N‐terminal region of the variable domain (VDI), has been implicated. A similar cryptic signal peptide sequence was identified within the precursor region of *R. reniformis* CLE pro‐peptides, although trafficking was not investigated (Wubben *et al*., 2015). Utilizing the same approach, it is possible to identify cryptic signal peptides within *R. reniformis* CEP precursors (Fig. S2, see Supporting Information) that have a strikingly high degree of conservation with the equivalent region in RrCLE1. However, further work is required to determine the route to the apoplast, which may be different from that used to deliver root‐knot nematode CEPs to their site of action. All CEP genes identified from both *M. hapla* and *Meloidogyne incognita* encode only a signal peptide, followed directly by the 15‐amino‐acid CEP domain (Bird *et al*., 2015). As for some other pharyngeal gland‐derived root‐knot nematode effectors (Vieira *et al*., 2011), these peptides are presumably delivered directly through the stylet into the apoplast, with post‐translational modifications occurring in the nematode. The CLE‐peptide effectors of *M. hapla* (Bird *et al*., 2015) similarly lack the pro‐domains that characterize both plant CLEs and those of cyst (Mitchum *et al*., 2012) and reniform (Wubben *et al*., 2015) nematodes, suggesting a more general divergence in effector delivery.

### Amino acids directly following CEP domains are highly non‐random in *R. reniformis* and plants

**Figure 4:**
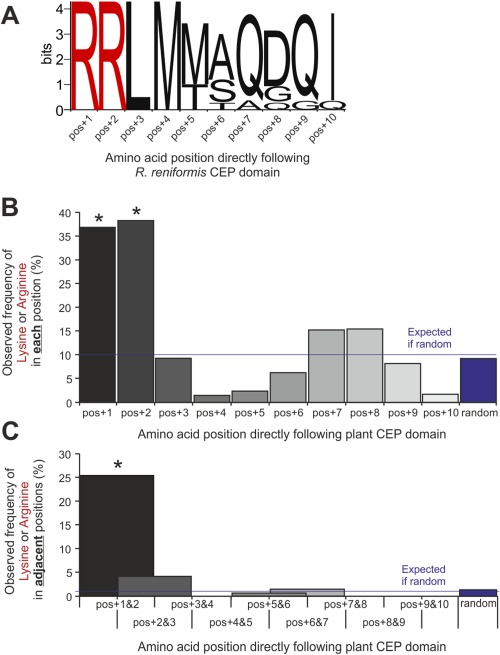
Non‐random single or di‐basic residues directly following nematode and plant C‐terminally‐encoded peptide (CEP) domains. (A) For all *Rotylenchulus reniformis* CEP sequences that contain more than one domain, the final domain is followed directly by a stop codon. However, all internal CEP domains, irrespective of domain sequence, are directly followed by a double arginine residue (*n* = 10). From 66 plant CEP genes across monocotyledonous and dicotyledonous species, amino acids directly following internal CEP domains are highly non‐random (*n* = 141, *χ* ^2^, *P < 0.0001) and are significantly enriched for single (B) and di‐basic (C) residues.

Ultimately, for CEPs to function, they require proteolytic processing to release the active domain from a pro‐protein. Like many plant peptide hormones, CEPs in plants are processed in the plant apoplast. Di‐basic residues adjacent to active domain motifs of plant pro‐peptide hormones are common in CLEs (DeYoung and Clark, 2001), PHYTOSULFOKINEs (PSKs) (Yang *et al*., 2001) and RAPID ALKALINIZATION FACTOR peptides (RALFs) (Matos *et al*., 2008); indeed, for AtRALF1, a double arginine is required for correct processing and function (Matos *et al*., 2008). For all cloned *R. reniformis* CEPs with more than one CEP domain, internal domains are directly followed by a double arginine as part of the consensus sequence RRLM (Fig. 4A). The AtS1P subtilisin which cleaves AtRALF23 has the canonical cleavage site RRIL (Srivastava *et al*., 2009). Therefore, the presence of single and di‐basic residues directly following the internal domains was also assessed for plant CEPs that contain more than one CEP domain. From 66 plant CEP genes across monocotyledonous and dicotyledonous species, amino acids directly following internal CEP domains are highly non‐random (*n* = 141, *P* < 0.0001) and are significantly enriched for both single and di‐basic residues (Fig. 4B,C), perhaps indicating that this is a common feature of all multi‐domain CEPs, not just those in animals.

Given that single and di‐basic residues are enriched directly following internal plant CEP domains, it is clear that the host machinery required to process nematode multi‐domain CEPs is already present in the apoplast, irrespective of their route to that compartment. Both animals and plants utilize di‐basic residues as cleavage sites; it is therefore possible that nematode CEPs are either processed in the animal and released as functional peptides or secreted as pro‐peptides and processed in the plant apoplast.

### *Rotylenchulus reniformis CEP1* encodes a biologically active peptide hormone

**Figure 5:**
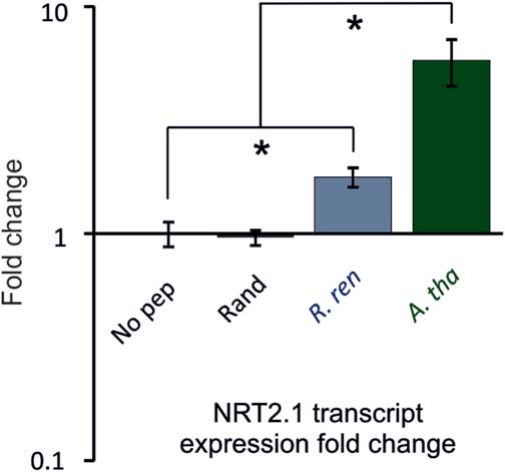
Exogenous application of synthetic *Rotylenchulus reniformis* CEP1.1 domain peptide up‐regulates nitrate transporter *AtNRT2.1* mRNA. Synthetic peptides corresponding to the RrCEP1.1 (*R. ren*) and AtCEP5 (*A. tha*) C‐terminally‐encoded peptide (CEP) domains at 1 μm significantly increase the transcript abundance of *Arabidopsis NRT2.1* compared with the no‐peptide control (No pep) (*n* = 3–4, *P < 0.05). No such effect is observed with an equimolar concentration of a randomized RrCEP1.1 peptide (Rand).

The current paradigm suggests that CEPs in plants have evolved as part of an adaptive response to nitrogen stress (Tabata *et al*., 2014); therefore, the ability of a CEP domain encoded by *RrCEP1* to up‐regulate the expression of an *Arabidopsis* nitrate transporter (*NRT2.1*) was tested. Synthetic peptides corresponding to the *Arabidopsis* CEP5 domain (AtCEP5, positive control) and to the first domain in RrCEP1 (RrCEP1.1) with hydroxyprolines in positions 4 and 11 were exogenously applied to *Arabidopsis* seedlings in plant growth medium. A randomized RrCEP1.1 peptide acted as a negative control. RrCEP1.1 significantly up‐regulated *NRT2.1* expression (Fig. 5, P < 0.05, *n* = 4), a response similar to, but lower in magnitude than, that induced by the endogenous plant CEP (AtCEP5), thus indicating that a CEP effector of *R. reniformis* encodes a biologically active peptide hormone. No effect was observed with the randomized RrCEP1.1. Up‐regulation of host nitrate transport provides a plausible benefit to both plant and animal.

### The dual roles of CEPs

**Figure 6:**
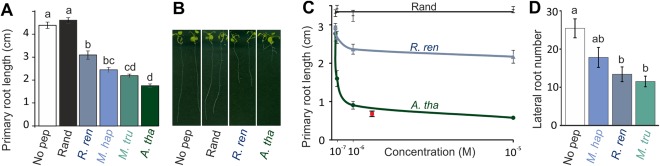
Exogenous application of synthetic *Rotylenchulus reniformis* CEP1.1 domain peptide affects root development in a dose‐dependent manner. (A) Synthetic peptides corresponding to the C‐terminally‐encoded peptide (CEP) domains of RrCEP1.1 (*R. reniformis*, *R. ren*), AtCEP5 (*Arabidopsis thaliana*, *A. tha*), MhCEP2 (*Meloidogyne hapla*, *M. hap*) and MtCEP1.1 (*Medicago truncatula*, *M. tru*) (1 μm) significantly reduce the primary root length of *A. thaliana* compared with a randomized RrCEP1.1 (Rand) or a no‐peptide control (No pep) at 15 days post‐germination (*n* = 21–27, lower case letters indicate homogeneous subsets, *P* < 0.001 accounting for multiple *t*‐tests; error bars indicate standard error of the mean). (B) Representative primary root length at 15 days post‐germination. (C) Increasing concentrations of both RrCEP1.1 and AtCEP5 increase the magnitude of the response. No response is observed for the randomized peptide, even at 10 μm. Combined application of RrCEP1.1 and AtCEP5 at equimolar concentrations (red, 1 μm each) does not reduce the effect of AtCEP5, suggesting that *R. reniformis* CEPs are not refractory and do not block the CEP receptor when in competition with endogenous plant CEPs, but function similarly to reduce the cell division rate. (D) RrCEP1.1 and MtCEP1.1, but not MhCEP2, reduce the lateral root number on *Medicago truncatula* compared with wild‐type (*n* = 16–23, lower case letters indicate homogeneous subsets, *P* < 0.002 accounting for multiple *t*‐tests; error bars indicate standard error of the mean).

CEPs also inhibit root proliferation by limiting the rate of cell division (Delay *et al*., 2013). Utilizing the well‐established system of reduction in *Arabidopsis* primary root length as a proxy for the inhibition of cell division, we were able to demonstrate that the RrCEP1.1 domain, the *M. hapla* CEP2 (MhCEP2) and the *Md. truncatula* CEP1.1 (MtCEP1.1) inhibit cell division, similarly to *Arabidopsis* CEP5 (Fig. 6A,B, *P* < 0.001 accounting for multiple *t*‐tests, *n* = 21–27). No such inhibition was observed with a randomized RrCEP1.1 peptide (Fig. 6A). In addition, the plant response to both RrCEP1.1 and AtCEP5 is dose dependent, as expected for a bona fide hormone ligand (Fig. 6C). No effect of increasing concentration was observed with the randomized RrCEP1.1 (Fig. 6C). Substituting canonical hydroxyprolines in positions 4 and 11 with Pro markedly reduced the magnitude of the host response for both AtCEP5 and RrCEP1.1 (Fig. S3, see Supporting Information). A similar requirement of hydroxyprolines in positions 4 and 11 of CEP peptides has been described previously (Delay *et al*., 2013, Imin *et al*., 2013), but it is not clear whether CEP domains lacking this hydroxylation are affected in receptor binding (Tabata *et al*., 2014), signal transduction or folding (Bobay *et al*., 2013). The inclusion of both RrCEP1.1 and AtCEP5 at equimolar concentrations produced the same effect as AtCEP5 alone (Fig. 6C), suggesting that RrCEP1.1 with hydroxyprolines in positions 4 and 11 is not refractory and does not block the CEP receptor when in competition with endogenous plant CEPs, but functions similarly to reduce the cell division rate.

**Figure 7:**
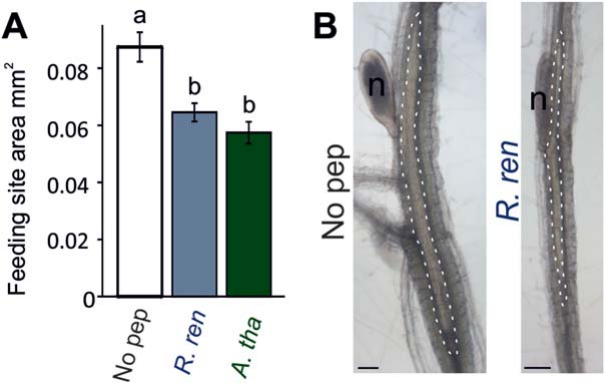
Allowing non‐C‐terminally‐encoded peptide (CEP)‐containing nematodes to infect in a CEP‐rich environment is detrimental to the size of the feeding site produced. (A) The size of the feeding site produced by *Heterodera schachtii* on *Arabidopsis thaliana* at 15 days post‐infection is significantly reduced when induced in the presence of 1 μm exogenous RrCEP1.1 (*R. ren*) or AtCEP5 (*A. tha*) (*n* = 45–59, lower case letters indicate homogeneous subsets, *P* < 0.0005 accounting for multiple *t*‐tests; error bars indicate standard error of the mean). (B) Representative syncytial size comparison between no‐peptide control (No pep) and RrCEP1.1 (n, nematode; syncytial boundaries are indicated by the broken white line).

Exploring CEP loss of function in *R. reniformis* is technically intractable; the efficacy of nematode gene knockdown by RNA interference (RNAi) is complicated by large gene families and exacerbated by redundancy, whereas the lack of a known host CEP receptor for the inhibition of cell division phenotype (Tabata *et al*., 2014) precludes the use of host mutants in the CEP response. During the induction of cyst nematode syncytia, a cell division event occurs in adjacent cells prior to their incorporation into the expanding feeding site (de Almeida Engler and Gheysen, 2013; Golinowski *et al*., 1996). Treatment of infected *Arabidopsis* roots with the mitotic inhibitor oryzalin prevented the division of neighbouring cells, resulting in smaller, narrower syncytia than in untreated roots (de Almeida Engler *et al*., 1999). Therefore, it might be expected that syncytium size is reduced in a CEP‐rich environment. The application of CEPs in a CEP‐negative state is required, given that the application of CEPs in an already CEP‐positive environment (*R. reniformis* infection of wild‐type *Arabidopsis*), either by exogenous application or over‐expression, is unlikely to be informative. Thus, CEP gain of function was explored in the cyst nematode species *Heterodera schachtii*. *Heterodera schachtii* forms a syncytial feeding site that involves cell division prior to incorporation, is able to infect *Arabidopsis* and, based on current knowledge, does not contain CEPs. These nematodes were therefore allowed to infect an *Arabidopsis* host in the presence of exogenous CEP peptide. When either RrCEP1.1 or AtCEP5 was included in the growth medium, syncytia induced by *H. schachtii* were significantly smaller than those induced in roots with no peptide (Fig. 7A,B, *P* < 0.0002, *n* = 45–59). This may indicate that an apparent cost associated with the production of CEPs to up‐regulate nitrate transporter expression is a reduced ability to create a large feeding site. Consistent with this, formation of the simple *R. reniformis* syncytium does not involve hyperplasia of cells (Robinson, 2007) and additional lateral cell layers do not appear to be incorporated (Jones and Dropkin, 1975). The syncytium typically comprises just a single layer of cells in the pericycle (Agudelo *et al*., 2005). Importantly, this apparent ‘trade‐off’ would not be valid for the other CEP‐containing nematode group (the root‐knot nematodes), as progression through the cell cycle to cytokinesis is inhibited in the normal formation of their feeding sites, the giant cells. An important consideration for both groups of CEP‐containing nematodes is the number of individuals required to produce a host response.

Relative to the effects of AtCEP5, both *NRT2.1* transcript up‐regulation and inhibition of root growth are similarly smaller in magnitude in response to RrCEP1.1. Although RrCEP1.1 and AtCEP5 differ by only three amino acids, CEPs in both *A. thaliana* and *R. reniformis* are within large gene families. It will be interesting to explore whether other *R. reniformis* CEPs are more effective at either of the dual roles for this particular plant species, or on any of its >300 host species (Robinson *et al*., 1998). RrCEP1.1, but not MhCEP2, modified root architecture in an additional host species, *Md. truncatula* (Fig. 6D), yet both were functional in *A. thaliana*, providing an indication that nematode CEPs may vary in efficacy depending on the host.

*Rotylenchulus reniformis* has one of the shortest life cycles amongst sedentary PPNs (Robinson *et al*., 1998), where eggs deposited by females hatch and, because of their lack of macro‐mobility, often re‐infect the same host, resulting in a single plant actively supporting thousands of closely related individuals. Thus, the presence of CEPs in this syncytial‐forming species may represent an adaptation to obligate dependence on host survival of individuals and their progeny for up to 10 generations (Barker *et al*., 1994; Karam *et al*., 2006). We hypothesize that CEPs of *R. reniformis* may contribute to a reduced syncytial size, and increase host nitrate uptake, thus reducing the overall drain on the host during infection.

## Experimental Procedures

### Nematode growth and collection

*Rotylenchulus reniformis* (Linford & Oliveira) was maintained on cotton plants (*Gossypium hirsutum* cv. Coker 201) growing in a mix of 2 : 1 loam soil : sand in a glasshouse at 25–27 ºC with a 16‐h day length. Sedentary females were extracted from washed roots at 8–10 weeks post‐infection. Clean root tissue was cut into approximately 2‐cm lengths and subjected to a short 2‐s burst in a Waring blender. The contents were then passed over a series of sieves and nematodes collected from the 150‐ and 63‐μm sieves were cleaned by sucrose (40%, w/v) centrifugation. Nematodes were extensively washed in tap water and individual young adult females were collected and flash frozen in liquid nitrogen. J2s were hatched from eggs collected from infected roots. Washed root tissue was blended as above for 10 s and homogenized tissue was passed over 150‐, 63‐ and 25‐μm sieves. Eggs were collected from the 25‐μm sieve, cleaned by sucrose centrifugation, washed and sterilized for 20 min in an appropriate volume of hexadecyltrimethylammonium bromide (CTAB, 0.5 mg/mL, Sigma, Dorset, UK) containing 0.1% v/v chlorhexidine digluconate (Sigma) and 0.01% v/v Tween‐20, followed by three washes in sterile tap water. Eggs were allowed to hatch at room temperature in water over a 25‐μm mesh. J2 nematodes were collected daily from under the 25‐μm mesh, cleaned as described above and flash frozen.

### RNAseq and transcriptome assembly

Total RNA was extracted from three biological replicate samples each of sedentary female and J2 nematodes using an RNeasy Mini kit (Qiagen, Manchester, UK) according to the manufacturer's instructions with an on‐column DNase I digestion. RNA quality was determined using a Bioanalyser (Agilent Technologies, Santa Clara, CA, USA). Samples with an RNA Integrity Number (RIN) > 8 were employed for library construction and sequencing using the service provided by The Genome Analysis Centre (TGAC, Norwich, UK), as described previously (Eves‐van den Akker *et al*., 2014a). Raw reads, trimmed of adapter sequences and low‐quality bases (*q* < 30), were assembled *de novo* using Trinity (Grabherr *et al*., 2011) with default parameters.

### Identification and cloning of *R. reniformis* CEP genes

*Rotylenchulus reniformis* transcripts encoding CEP‐like sequences were identified from the assembled transcriptome by regular expression search as described previously (Eves‐van den Akker *et al*., 2014a). Three transcripts encoding CEP‐like sequences were identified (Dryad accession doi:10.5061/dryad.q8h75). Forward (ATGAAATTGACCTTAATTTTGATGCT) and reverse (TCAATGTCCATCTCCAGGAGAATG) oligonucleotide primers were designed to clone the genomic DNA sequence corresponding to comp45145_c2_seq7. DNA was isolated from J2s, collected as above and extracted as described previously (Eves‐van den Akker *et al*., 2014b). Sequences of interest were amplified using Phusion Hi‐fidelity proofreading polymerase, following the manufacturer's instructions (New England Biolabs, Hitchin, Herts, UK). 5′ Adenosines were added to PCR products by incubation at 72 ºC with the addition of 1 µL BioTaq DNA polymerase (Bioline, London, UK), and ligated into the pGEM‐T Easy vector (Promega, Southampton, UK). Positive clones were confirmed by sequencing. Amino acid sequences were deduced by similarity to sequences in the transcriptome assembly.

### Phylogenetic and motif analyses

Cloned genomic sequences which encoded complete open reading frames, were unique at the protein level (or, where identical, contained considerably different introns) and were different in total gene length were deemed to arise from unique loci and were used to construct a preliminary phylogeny of the gene family. Complete genomic sequences were aligned and refined using MUSCLE (Edgar, 2004), and used to construct a Bayesian phylogeny of the gene family employing 300 000 generations in TOPALi (Milne *et al*., 2009). Based on the above criteria, highly similar sequences were collapsed into a single representative displayed in Fig. 1. CEP domain motif plots and di‐basic residue plots were generated using MEME (Bailey *et al*., 2009). The putative ‘cryptic signal peptide’ was identified by analysing a 50‐base‐pair sliding window across the protein sequence of RrCEP1 using SignalP v4.0 (Petersen *et al*., 2011)

### Differential expression and *in situ* hybridization

Trimmed RNAseq reads were mapped back to the transcriptome using tophat2 (Kim *et al*., 2013). Read counts were TMM normalized, and differentially expressed transcripts were identified using the trinity wrapper scripts for EdgeR, specifying a minimum *P* value of 0.001 and a minimum fold change of four. *In situ* hybridization was carried out with sedentary‐stage adult females of *R. reniformis*, fixed and extracted from the roots of cotton plants according to previously described methods (Eves‐van den Akker *et al*., 2014b). Cut nematodes were treated with proteinase K (2 mg/mL) for 1 h at room temperature and then the *in situ* hybridization protocol was continued from the dehydration step, as described by de Boer *et al*. (1998). Single‐stranded, digoxygenin‐labelled hybridization probes, 140 bp in length, were synthesized as described previously (Eves‐van den Akker *et al*., 2014b) using forward (CTGGACGGCTTTTAGTGCAC) and reverse (CAGTCAGAACGCGTGCAAAA) primers to generate sense and anti‐sense probes, respectively.

### Plant growth and root analysis

Surface‐sterilized seeds of *Arabidopsis thaliana* (Col‐0) were stratified for 1–2 days and sown onto 9‐cm‐diameter Petri plates containing 25 mL of half‐strength Murashige and Skoog (MS) medium adjusted to pH 5.7 and solidified with 1% phytagel. Where indicated, synthetic peptides corresponding to AtCEP5 (DFR‐hyP‐TTPGHS‐hyP‐GIGH), MtCEP1.1 (AFQ‐hyPTTPGNS‐hyPGVGH), MhCEP2 (AFR‐hyPTAPGHS‐hyPGVGH), RrCEP1.1 (AFK‐hyP‐TTPGHS‐hyP‐GAGH) or its randomized version RrCEP1.1rand (TA‐hyP‐GTHGP‐hyP‐SFAKGH) at 1 mm in 50 mm sodium phosphate buffer, pH 6, were added to the medium immediately before pouring to achieve the desired final concentration. Plates were held vertically in a growth chamber (Microclima, Snijders Scientific, Tilburg, The Netherlands) at 20 ºC with a 16‐h day length at 15 μmol/m^2^/s photosynthetically active radiation and 70% humidity. Plates were imaged using an HP scanner and primary root lengths were calculated from saved images using Image‐Pro Analyser v7 (MediaCybernetics, Rockville, MD, USA). Significant differences in root lengths between treatments were determined by analysis of variance (ANOVA) with a Tukey *post hoc* test. *Medicago truncatula* seedlings were prepared as above, but grown on Fahraeus medium with no nitrogen (according to Imin *et al*., 2013) under 11 μmol/m^2^/s of photosynthetically active radiation.

### Nematode infection and feeding site measurement

Seedlings of *Arabidopsis* were grown as described above with the addition of 1% sucrose to the medium. J2s of the beet cyst nematode *H. schachtii* were hatched from cysts, sterilized as described previously (Davies *et al*., 2012) and then resuspended in 0.01% Tween‐20 at a concentration of approximately one nematode/µL. At 12 days post‐imbibition, three root tips per plant were each inoculated with 20 µL of nematode suspension with two plants per plate. Infection points were covered with GF/A paper (Whatman, Maidstone, Kent, UK) for 2 days to facilitate invasion. All root lengths harbouring syncytia at 15 days post‐infection (dpi) were excised, mounted on microscope slides and images were captured using an Olympus, Southend, Essex, UK BH2 microscope with a MicroPublisher 3.3 RTV camera and Q‐Capture Pro software (QImaging, Surrey, BC, Canada). Syncytium sizes were estimated from the projected cross‐sections and measured using Image‐Pro Analyser v7 (MediaCybernetics). At least 45 individual syncytia per treatment were measured for plants growing on unsupplemented medium or on plates containing 1 μm AtCEP5 or 1 μm RrCEP1.1.

### RNA extraction, cDNA synthesis and quantitative reverse transcriptase‐polymerase chain reaction (qRT‐PCR) analysis

Total RNA was isolated at 16 days post‐imbibition from the pooled roots of six *Arabidopsis* seedlings per treatment using an RNeasy Plant Mini Kit (Qiagen) according to the manufacturer's instructions. An on‐column DNase treatment was carried out. cDNA was prepared from 500 ng of RNA using SuperScript II reverse transcriptase (Invitrogen, Carlsbad, CA, USA) and an anchored oligo(dT) primer according to the manufacturer's instructions. qRT‐PCR was carried out on the resulting cDNA using Brilliant III SYBR® Green Master Mix (Agilent Technologies) to determine the expression level of the high‐affinity nitrate transporter *NRT2.1* (At1g08090). qRT‐PCR conditions were as follows: 95 ºC for 3 min, followed by 40 cycles of 95 ºC for 30 s, 60 ºC for 30 s and 72 ºC for 30 s. Fluorescence data were collected at the end of the annealing phase. Primer pairs for *NRT2.1* (FOR, 5′‐TCTTTTGGGTCCCCGTTACG‐3′; REV, 5′‐CGCCAGGCAAAAACCAATCA‐3′) and *Elongation Factor 1‐α* At5g60390 (FOR, 5′‐GACAGGCGTTCTGGTAAGGA‐3′; REV, 5′‐GCTTGGTTGGGGTCATCTTA‐3′) had amplification efficiencies of 90%–110%. Relative expression was determined by the efficiency‐corrected ΔΔC_T_ method from a minimum of three biological replicates per treatment, each with three technical replicates and using elongation factor for normalization.

### Accession numbers

Raw RNAseq reads are available under SRA accession PRJEB8325. Transcripts encoding CEP‐like sequences identified in the transcriptome, all cloned CEP sequences and the CEP phylogeny .tre file are available from the Dryad Digital Repository: http://dx.doi.org/10.5061/dryad.q8h75

## Supporting information

Additional Supporting Information may be found in the online version of this article at the publisher's website:

**Fig. S1** Insight into the genomic organization of *Rotylenchulus reniformis* C‐terminally‐encoded peptides (CEPs). Schematic diagram to show the organization of two *RrCEP* genes cloned in a tandem array as a single amplification product.

**Fig. S2** Amino acid features of RrCEP1.1. Using a 50‐bp sliding window across the full length of RrCEP1.1, two putative signal peptide cleavage sites are identified (SignalP v4.0): one at the N‐terminus (blue) and a second prior to the first (internal) C‐terminally‐encoded peptide (CEP) domain. Signal peptide cleavage sites were also identified in equivalent positions for the other RrCEPs. CEP domains are highlighted in red, di‐basic residues in orange and a conserved KND motif of unknown function present prior to CEP domains in green.

**Fig. S3** Comparison of the activity of RrCEP1.1 and AtCEP5 with and without hydroxyprolines. Substituting canonical hydroxyprolines in positions 4 and 11 with proline markedly reduced the magnitude of the host response for both AtCEP5 and RrCEP1.1 (Student's *t*‐test, *n* = 36–40, error bars indicate standard error of the mean).
