# Supplementary figures and images for: Functional C‐TERMINALLY ENCODED PEPTIDE (CEP) plant hormone domains evolved de novo in the plant parasite Rotylenchulus reniformis
Source: Mol Plant Pathol. 2016 Jun 6;17(8):1265–75. doi: 10.1111/mpp.12402 (PMC5103176; doi:10.1111/mpp.12402)

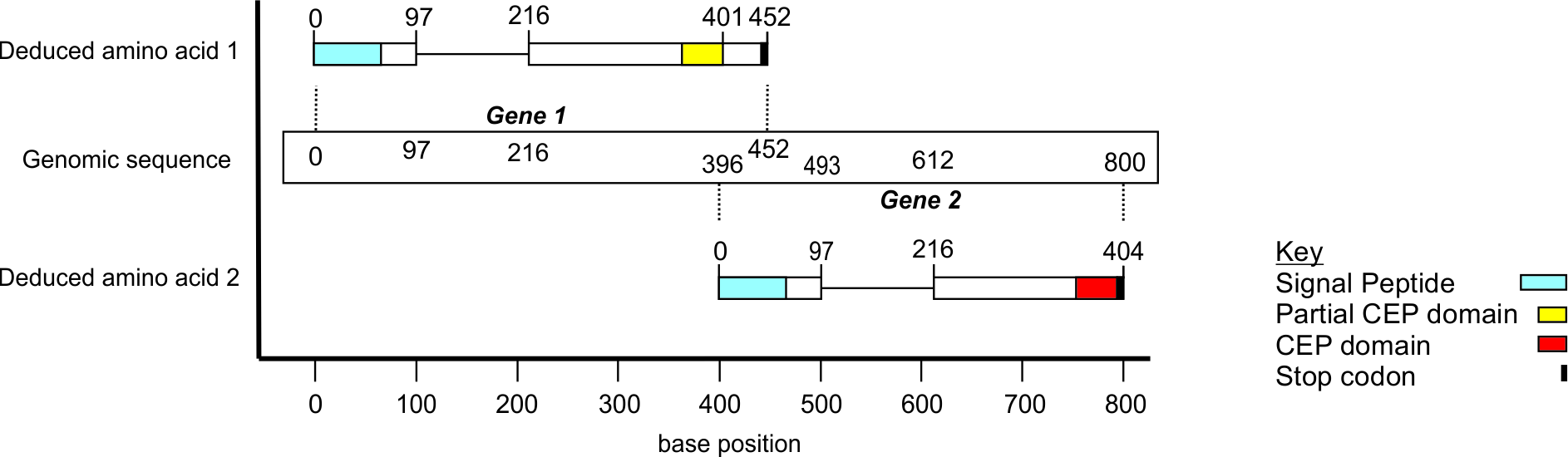

Supplement: Supplementary file 1 — Fig. S1 Insight into the genomic organization of Rotylenchulus reniformis C‐terminally‐encoded peptides (CEPs). Schematic diagram to show the organization of two RrCEP genes cloned in a tandem array as a single amplification product. [file MPP-17-1265-s001.tif]

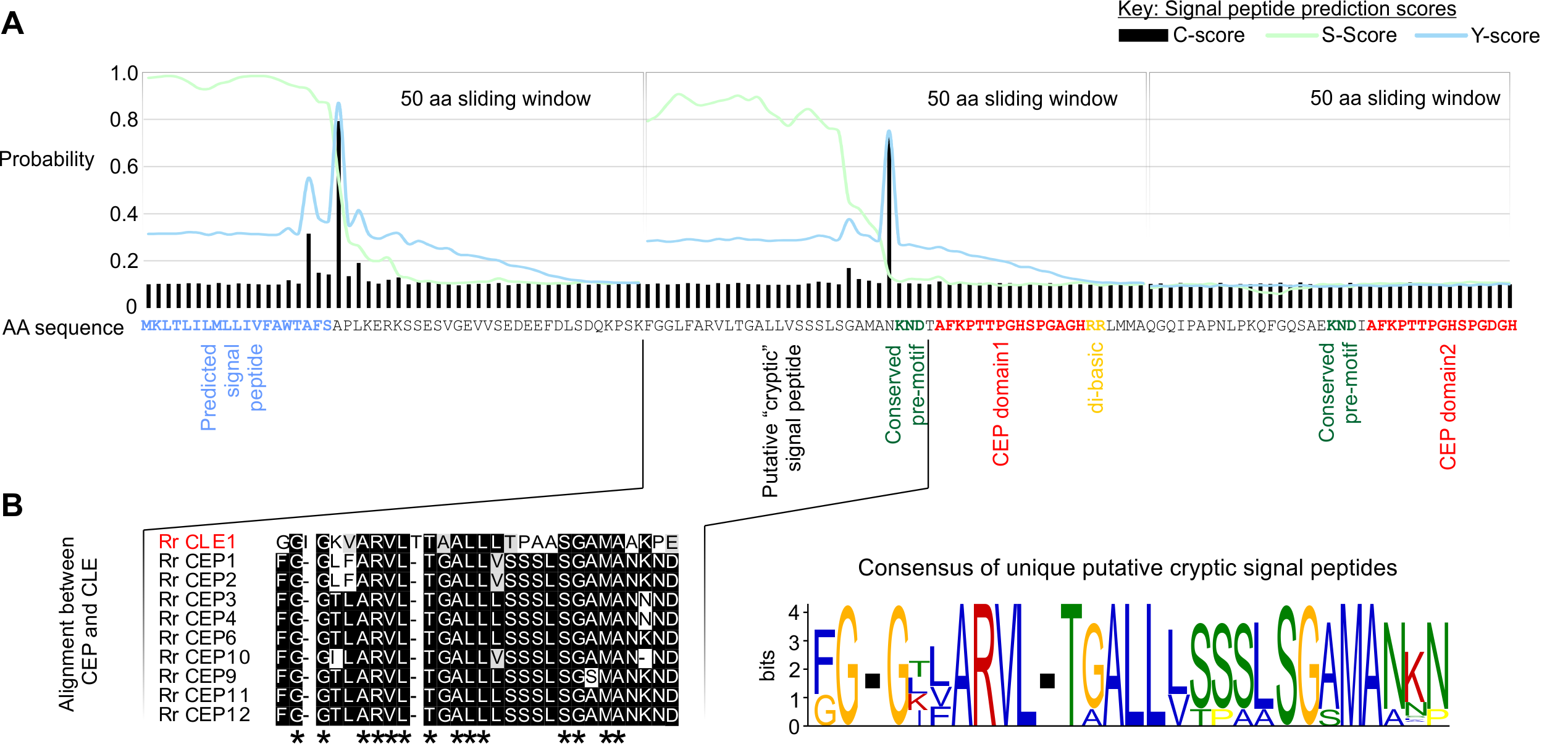

Supplement: Supplementary file 2 — Fig. S2 Amino acid features of RrCEP1.1. Using a 50‐bp sliding window across the full length of RrCEP1.1, two putative signal peptide cleavage sites are identified (SignalP v4.0): one at the N‐terminus (blue) and a second prior to the first (internal) C‐terminally‐encoded peptide (CEP) domain. Signal peptide cleavage sites were also identified in equivalent positions for the other RrCEPs. CEP domains are highlighted in red, di‐basic residues in orange and a conserved KND motif of unknown function present prior to CEP domains in green. [file MPP-17-1265-s002.tif]
