# Supplementary material for: Functional C‐TERMINALLY ENCODED PEPTIDE (CEP) plant hormone domains evolved de novo in the plant parasite Rotylenchulus reniformis
Source: Mol Plant Pathol. 2016 Jun 6;17(8):1265–75. doi: 10.1111/mpp.12402 (PMC5103176; doi:10.1111/mpp.12402)
Supplement: Supplementary file 3 — Fig. S3 Comparison of the activity of RrCEP1.1 and AtCEP5 with and without hydroxyprolines. Substituting canonical hydroxyprolines in positions 4 and 11 with proline markedly reduced the magnitude of the host response for both AtCEP5 and RrCEP1.1 (Student's t‐test, n = 36–40, error bars indicate standard error of the mean). [file MPP-17-1265-s003.pdf]

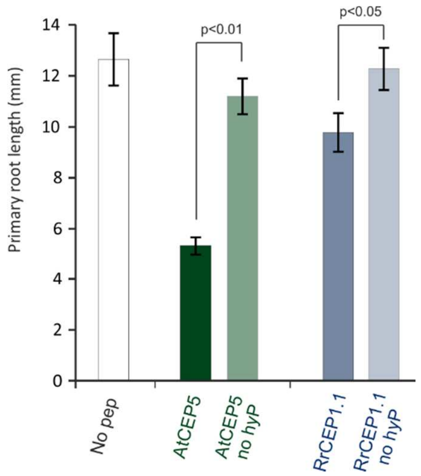

Figure S3. Comparison of activity of RrCEP1.1 and AtCEP5 with and without hydroxyprolines. Substituting canonical hydroxyprolines in positions 4 and 11 with proline markedly reduced the magnitude of host response for both AtCEP5 and RrCEP1.1 (Student's T-test, n=36-40, error bars indicate standard error of the mean).
